# An Evaluation of Emergency Medicine Core Content Covered by Podcasts

**DOI:** 10.5811/westjem.2022.11.57717

**Published:** 2023-01-11

**Authors:** Jeffrey Riddell, Scott Kobner, Gabriel Padilla

**Affiliations:** * Keck School of Medicine of USC, Department of Emergency Medicine, Los Angeles, California; ‡ Alpert Medical School of Brown University, Department of Emergency Medicine, Providence, Rhode Island

## Abstract

**Introduction:**

Podcasts are used broadly for learning in emergency medicine (EM); however, there is concern about the potential for knowledge gaps for learners who rely on podcasts for their learning. The extent to which EM podcasts cover the core curriculum of EM is not known; thus, we sought to quantify the extent to which podcasts represent the core content of our specialty.

**Methods:**

We performed a retrospective review of all EM podcast episodes published in 2019. All podcasts were given credit for the content they covered as it related to the 2016 American Board of Emergency Medicine (ABEM) Model of Clinical Practice in Emergency Medicine (EM Model). The primary outcome was a description of how podcasts represented the ABEM EM Model content topics compared to the topic representation of the ABEM Qualifying Exam.

**Results:**

We included 54 unique EM podcast programs and 1,193 podcast episodes. They covered 2,965 total EM Model core content topics. The topics most covered were “other” (which includes interpersonal skills and professionalism), procedures, and signs and symptoms. Musculoskeletal, hematology, and environmental each accounted for less than 1% of all topics covered. Almost three-quarters of podcast episodes covered other core competencies of the practice of EM.

**Conclusion:**

Podcasts had a broad yet imbalanced coverage of the ABEM EM Model core content subtopics in 2019, with a notable coverage of other core competencies of the practice of EM. Learners, educators, and scholars should be mindful of these gaps and focus future work on exploring how podcasts should best be used in EM education.

## INTRODUCTION

Medical students, residents, and practicing physicians increasingly use podcasts for their education, with some preferring podcasts to textbooks and journal articles.^1^–^6^ Podcasts are an easy-to-use and engaging medium that provide learners with broad exposure to educational content and targeted learning opportunities.^7^,^8^ While educational podcasts are used in many medical specialties,^9^–^13^ podcasting is particularly popular in emergency medicine (EM). To date, EM has the largest number of active podcasts, podcast episodes, and hours of content.^14^

While some hail the rise of podcasts as the “Netflix” of medical education,^15^ others sound notes of caution. In addition to concerns over quality,^16^,^17^ some argue that online educational resources lack the comprehensiveness of textbooks and other commonly used curricula designed to impart the breadth of core-content knowledge dictated by the American Board of Emergency Medicine (ABEM) Model of the Clinical Practice of Emergency Medicine (EM Model).^18^,^19^ Thus, learners who rely primarily on these resources may have knowledge gaps due to over- or under-representation of topics.^20^

This begs the question, however, of whether the amalgam of EM podcasts should be comprehensive. If podcasts are poised to “fundamentally reimagine medical education” in the next decade,^21^ then we must wrestle with their role and purpose. Although some podcasts have been designed specifically to cover EM core content,^22^–^24^ should we ask podcasts to cover the breadth of an EM education? And if so, can they? It is unclear the extent to which even the core content-focused podcasts cover the breadth of the specialty. Further, within EM core-content podcasts, are they disproportionately covering topics that are probably best taught in a visual (eg, dermatology) or kinesthetic (eg, procedures) format? Or should we ask instead of podcasts that they bring multi-specialty expertise to controversial topics,^25^ model clinical reasoning and diagnosis,^26^ analyze recent clinical literature,^27^ focus on mindset and mental performance,^28^ tackle gender equity in medicine,^29^ or carry forward conversations about racism in medicine?^30^–^32^

Given trainees’ current dependence on these resources, and the ways in which many learners listen by absorbing whatever topic is pushed to them in the queue,^7^ our responses to these debates should be informed by a comprehensive understanding of what podcasts are currently covering. This knowledge will begin to help educators make thoughtful recommendations to their learners, help podcast creators see the domains they may have been over- or under-emphasizing, and help scholars make educated arguments about the role and purpose of podcasts in EM education. The only existing studies looking at the comprehensiveness of online educational resources have focused on blogs,^19^,^20^ leaving a gap in our understanding of the content podcasts provide. Thus, we sought to quantify the extent to which podcasts represent the core content of our specialty.

## METHODS

### Study Design

We performed a retrospective review of all available episodes from 54 EM educational podcasts posted online in 2019. We followed the approach used in a previous study evaluating blog content, and we mapped the content of all included podcast episodes to the 2016 ABEM EM Model content topics.^18^,^19^ Our local institutional review board deemed the study exempt.

### Podcast Selection

As there was no updated listing of all EM podcasts, we used a three-step process to identify all EM podcasts that released episodes in 2019. First, we included all accessible podcasts documented in a recent study that relied on a web search to identify podcasts in every medical specialty (including EM).^14^ This yielded 32 EM podcasts for inclusion. Next, we searched the Social Media Index (SMI) on March 31, 2020, to find podcasts that we had not yet included. The SMI is a website that lists EM and critical care blogs and podcasts, ranking them based on their impact.^33^,^34^ The SMI yielded 39 additional podcasts. Finally, we emailed a group of seven EM podcast creators for their content expertise. We showed them our existing list and asked them to recommend any podcast we might have missed. They recommended 14 additional podcasts. From a total of 85 podcasts, we then excluded duplicates (those on both the web search study and the SMI), podcasts not focused on EM, and podcasts without any episodes during 2019.

**Population Health Research Capsule**

What do we already know about this issue?

*Emergency medicine (EM) trainees utilize podcasts extensively for learning.*

What was the research question?

*Does the representation of core content topics in EM podcasts differ significantly from the* American Board of Emergency Medicine (*ABEM) qualifying exam?*

What was the major finding of the study?

*Podcasts had an imbalanced yet broad coverage of ABEM EM Model core content subtopics.*

How does this improve population health?

*Listeners, educators, and podcast creators should be mindful of these gaps and consider which parts of the core curriculum of our specialty are best suited to audio learning.*

**Figure 1:**
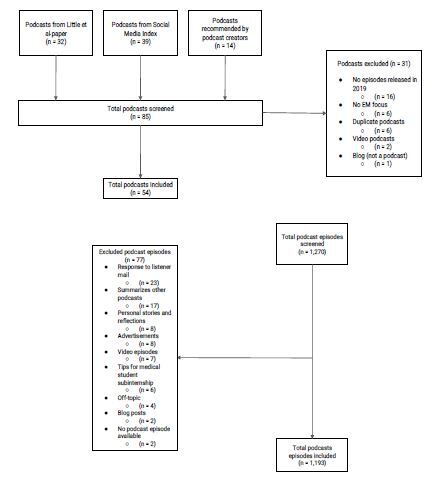
Podcast selection.

The 54 remaining podcasts yielded 1,270 unique podcast episodes. We excluded individual episodes if they were a video/vodcast, mailbag-response episode, summarized another podcast, largely advertisements, or focused on personal stories. A total of 77 episodes were excluded, leaving 1,193 for our analysis (Figure 1). The list of included podcasts is available as eAppendix A.

### Measurement methods

We used the 2016 ABEM EM Model clinical categories and subtopics to define EM core content.^18^ The 2016 model was used because the updated 2019 ABEM EM Model was not publicly available at the start of the study. There are a total of 20 broad categories in the EM Model, which are each divided into subtopics. We defined each category within the EM Model as a core content topic.

One author (GP) evaluated each podcast episode for EM Model, core content coverage by reviewing the podcast episode title and show notes. If show notes were unavailable, the author listened to the podcast. If a podcast covered any EM Model core content topic in an educational manner, we documented that podcast as having covered its corresponding EM Model core content category. We gave podcasts credit for as many EM Model core content topics as were mentioned. In other words, a single podcast could have covered multiple EM Model core content topics.

If a topic was merely mentioned incidentally but not expanded upon in an educational manner, the podcast episode was not credited with covering that topic. For example, if a podcast was discussing a cardiovascular diagnosis and then mentioned that the differential diagnosis included a gastrointestinal topic but did not describe it in any detail, then the podcast would only receive credit for the cardiovascular topic. However, if the gastrointestinal topic was described in further detail, then the podcast episode would be credited with covering that topic as well.

Throughout the coding process a second author (JR) reviewed and discussed any unclear podcast topic coverage until consensus was achieved. A third author (SK) then abstracted data from a random sample of 5% of podcasts and coded each podcast in a similar manner to the primary abstractor. Their inter-rater agreement was excellent (pooled k = 0.84). The inter-rater agreement broken down by each EM Model topic is available in eAppendix B.

### Outcomes

For the purpose of comparison given our primary focus on resident education, and in keeping with previous blog-based studies,^19^,^20^ we used the ABEM Qualifying Exam (QE) content specifications as the standard weighting of core content.^35^ The primary outcome was a description of how ABEM EM Model content topics were represented by EM podcasts from January 1–December 31, 2019 compared to the topic representation of the ABEM QE. Additional outcomes included the number of unique EM podcast episodes that covered each EM Model category.

### Data Analysis

We calculated proportional representation of ABEM categories covered by podcasts with associated 95% confidence intervals (CI). We also calculated differences between categorical representation in podcasts and the ABEM QE.

## RESULTS

The 54 unique EM podcasts yielded 1,193 included podcast episodes. They covered 2,965 EM Model topics, with a mean of 2.49 (SD 1.77) subtopics per podcast. Of all topics, those most commonly covered were as follows: “other” (29.1%, 95% CI 27.75–30.53); procedures (15.8%, 95% CI 14.54–16.96); and signs and symptoms (12.9%, 95% CI 11.76–14.01). When compared to their relative weight on the ABEM QE, the same topics were the only ones notably over-represented (Table 1). Musculoskeletal (0.7%, 95% CI 0.43–1.05), hematology (0.8%, 95% CI 0.52–1.17), and environmental (0.9%, 95% CI 0.54–1.21) each accounted for less than 1% of all topics covered. The most under-represented topics relative to their weight on the ABEM QE were abdominal/GI (−5.1%), trauma (−4.0%), respiratory (−3.7%), and cardiovascular (−3.5%).

The percentage of all podcast episodes that covered each ABEM topic are shown in Table 2. Almost three-quarters (72.4%, 95% CI 70.8–74.0) of podcast episodes covered other core competencies of the practice of EM, which was 70.4% more than the topic’s relative weight on the ABEM QE. Signs and symptoms and procedures were also covered on more than 32% and 39% of podcast episodes, respectively. While many topics were over-represented relative to the ABEM QE, relatively few were under-represented as a proportion of all podcast episodes, including abdominal/GI (−2.4%); head, eyes, ears, nose, and throat exam (−1.3%); musculoskeletal (−1.2%); and hematology (−0.9%).

## DISCUSSION

We found notable differences in how ABEM EM Model content topics were represented by EM podcasts compared to the topic representation of the ABEM Qualifying Exam. Overall, our data suggests imbalanced yet broad coverage of the core content of EM. The finding that, as a percentage of all podcast episodes, very few topics were proportionally under-represented relative to their ABEM QE weight speaks to the thorough coverage of core content that podcasts provided. This is in line with a previous study of blogs that also found five topics under-represented compared with the QE model.^20^

**Figure 2:**
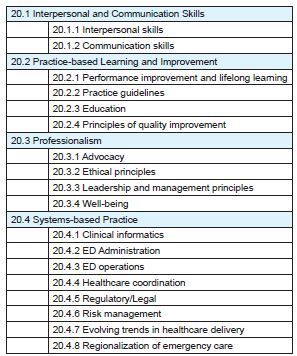
Subtopics under “20.0 Other Core Competencies of the Practice of Emergency Medicine” category in the 2016 ABEM EM Model. *ABEM*, American Board of Emergency Medicine; *EM*, emergency medicine; *ED*, emergency department.

When considering all topics covered, we found a range of proportional representation relative to the QE from +27% to −5%. The imbalance we found with podcasts is not strikingly dissimilar to previous studies of blog posts.^19^,^20^ The imbalance in podcasts, however, appears different from the imbalance found in the blog studies. While these studies also reported high proportions of signs and symptoms, procedures, and other competencies,^19^ we found a strikingly higher level of discussion of other core competencies of the practice of EM on podcasts. The subtopics for other core competencies of the practice of EM are shown in Figure 2.

The finding that other competencies accounted for 29% of all topics covered and was covered on 72% of all podcasts reveals a lot about the role podcasts may be filling in the EM educational milieu. Whereas a leading EM textbook has now relegated chapters on multiculturalism, bioethics, medicolegal issues, wellness, stress, and the impaired physician to its “Bonus Online Content,”^36^ it appears podcasts are elevating, or at least making more frequent, these discussions. There are several factors likely contributing to this discrepancy. First, it may be a function of time. As the first study highlighting imbalanced coverage of core content studied the landscape in 2013–2014,^19^ and the other between 2015–2017,^20^ it is possible that podcast creators have reacted to these studies and/or current events in creating content that addresses current pressing issues around subjects such as professionalism or systems-based practice.

Similarly, while some topics like “other” may be over-represented because they are covered by recently published, high-impact, primary literature articles, the topics that were under-represented might not have been featured in recent primary literature publications. For example, the EM Model subtopic “status epilepticus” made up 8.5% of the coverage for the nervous system category (data not shown). This is largely due to multiple podcast episodes in 2019 that discussed the ConSEPT and EcLiPSE trials following their publication in *Lancet* in May 2019.^37^,^38^ We did not, however, find similarly impactful trials published about “inter-departmental and medical staff relations” or “clinical decision support,” two of the most covered topics in the category “other.”

To address the questions raised in the introduction, our data suggests that learners who rely primarily on podcasts may have knowledge gaps due to over- or under-representation of topics. Given that most podcast listeners (including EM residents, attendings, and other healthcare professionals) indicate that they listen to podcasts to learn EM core content,^2^,^3^,^5^,^39^ it is possible that some learners may be unaware of the gaps in EM core content coverage. If they do not continue to use a broad range of resources for their education, they could miss out on the full breadth of knowledge necessary to pass the ABEM QE and be prepared for every patient who enters the ED. 40 Therefore, listeners, educators, and podcast creators should be aware of these gaps. Several podcasts have, however, been created to specifically cover the breadth of EM core content. Most notably, there is a podcast that covers every chapter of *Rosen’s Emergency Medicine* textbook.^22^,^41^

The question of whether the amalgam of EM podcasts should be comprehensive is a thornier one. While our data does not definitively answer that question, it does suggest that podcast creators are overwhelmingly bringing what they think is important into their audio conversations. It is likely that resident learners also listen to podcasts, and podcast producers create podcasts for reasons other than learning EM core content. Podcast users have various reasons for listening including to be entertained, to connect with the EM community, to be inspired, and to engage in targeted personalized learning.^5^,^7^ These important reasons for using podcasts do not rely on them to broadly cover all of EM core content. Rather, the benefit of podcasts in the EM educational space may have more to do with the depth that the format enables.

Podcasts offer long-form “deep dives” infused with immersive storytelling that can provide more gravitas than their written counterparts.^42^ The personal nature of audio allows the creator to stimulate the imagination of the listener using richly textured narratives and self-reflexive gestures, thereby influencing both motivation and cognition.^43^,^44^ In contrast to blogs and textbooks, the heightened intonations and subtle adjustments in volume, inflexion, and phrasing allowed by the spoken word can communicate emotions, create a sense of intimacy, and captivate audiences for extended periods.^8^,^42^,^45^ The format allows for conversations about workplace-based violence, compliance, litigation, reimbursement issues, burn-out, and diversity in ways that just aren’t possible with other educational media (blogs, textbooks, journal articles, etc).

Therefore, future work might focus less on ensuring that every core content subtopic gets covered by a podcast and more on understanding the content that is included in podcasts. It would be fruitful to explore why topics are chosen, how they stimulate emotions and imagination, how to make podcast learning more effective,^43^ how podcasts impact professionalism and systems-based practice and, ultimately, which parts of the core curriculum of our specialty are best suited to audio learning.

## LIMITATIONS

This study has many limitations. First, we evaluated only podcasts released in 2019, and it is possible that including podcast episodes released in prior years would reduce the gaps in coverage of EM core content. Further, impactful studies during the year of release could also have skewed the data. Second, podcasts were evaluated based mostly on the episode title and show notes. We were not able to listen to every podcast episode due to logistical time constraints. It is possible that EM Model core content could have been covered in actual audio podcast recordings without being outlined in the show notes, which could have led us to underestimate the extent of EM core content coverage by podcasts and perhaps points to the need for a standardized template for show notes.

Third, determining what constitutes a meaningful educational discussion of a subtopic was difficult. We attempted to remedy this by discussing all questionable educational points and achieving group consensus, including a large number of podcast episodes, and having a second abstractor review a random percentage of podcasts with excellent inter-rater reliability. Finally, our study was designed to simply evaluate how podcasts covered ABEM EM Model core content. We did not evaluate the depth or quality of coverage, nor did we explore podcast creators’ qualifications or their use of primary literature references. Future studies should continue to explore the issues affecting the quality of EM educational podcasts.^33^,^46^

## CONCLUSION

Podcasts had an imbalanced yet broad coverage of ABEM EM Model core content subtopics in 2019. We found imbalances in content representation compared to the weight given in the ABEM Qualifying Exam, with a strikingly higher level of discussion of other core competencies of the practice of emergency medicine on podcasts. Learners, educators, and scholars should be mindful of these gaps and focus future work on exploring how podcasts should best be used in EM education.

## Supplementary Information





## Figures and Tables

**Table 1:** Proportional representation of the American Board of Emergency Medicine EM Model topics covered in podcasts relative to the ABEM Qualifying Exam.

ABEM Topic	Relative weight on ABEM Qualifying Exam	Total number of podcast episodes that covered topic	Topic representation among all topics covered (n=2,965)	95% CI	Difference between topic representation among all topics covered and percentage of ABEM Qualifying Exam
Signs and symptoms	10%	382	12.9%	11.76 – 14.01	2.9%
Abdominal/GI	7%	55	1.9%	1.37 – 2.34	−5.1%
Cardiovascular	10%	192	6.5%	5.62 – 7.33	−3.5%
Cutaneous	3%	36	1.2%	0.82 – 1.61	−1.8%
Endocrine	5%	78	2.6%	2.06 – 3.20	−2.4%
Environmental	2%	26	0.9%	0.54 – 1.21	−1.1%
HEENT	4%	32	1.1%	0.71 – 1.45	−2.9%
Hematology	3%	25	0.8%	0.52 – 1.17	−2.2%
Immune system	2%	30	1.0%	0.65 – 1.37	−1.0%
Infectious disease	7%	112	3.8%	3.10 – 4.45	−3.2%
Musculoskeletal	3%	22	0.7%	0.43 – 1.05	−2.3%
Nervous system	6%	120	4.0%	3.35 – 4.74	−2.0%
OB/GYN	3%	31	1.0%	0.68 – 1.41	−2.0%
Psychobehavioral	2%	64	2.2%	1.64 – 2.68	0.2%
Renal and urogenital	3%	53	1.8%	1.31 – 2.26	−1.2%
Respiratory	7%	97	3.3%	2.64 – 3.90	−3.7%
Toxicology	4%	131	4.4%	3.70 – 5.14	0.4%
Trauma	9%	148	5.0%	4.23 – 5.76	−4.0%
Procedures	8%	467	15.8%	14.54 – 16.96	7.8%
Other	2%	864	29.1%	27.75 – 30.53	27.1%
Total	100%	2,965	100.0%		

*ABEM*, American Board of Emergency Medicine; *EM*, emergency medicine; *CI*, confidence interval; *GI*, gastrointestinal; *HEENT*, head, eyes, ears, nose, and throat; *OB/GYN*, obstetrics/gynecology.

**Table 2:** Proportional representation of individual podcast episodes that covered ABEM EM Model topics relative to the ABEM Qualifying Exam.

ABEM topic	Relative weight on ABEM Qualifying Exam	Total number of podcast episodes that covered topic	Percentage of podcasts that covered topic (n=1,193)	95% CI	Difference between percentage of podcasts that covered topic and percentage of ABEM Qualifying Exam
Signs and symptoms	10%	382	32.0%	29.8 – 34.2	22.0%
Abdominal/GI	7%	55	4.6%	3.4 – 5.8	−2.4%
Cardiovascular	10%	192	16.1%	14.2 – 18.0	6.1%
Cutaneous	3%	36	3.0%	2.1 – 4.0	0.0%
Endocrine	5%	78	6.5%	5.2 – 7.9	1.5%
Environmental	2%	26	2.2%	1.4 – 3.0	0.2%
HEENT	4%	32	2.7%	1.8 – 3.6	−1.3%
Hematology	3%	25	2.1%	1.3 – 2.9	−0.9%
Immune system	2%	30	2.5%	1.6 – 3.4	0.5%
Infectious disease	7%	112	9.4%	7.8 – 11.0	2.4%
Musculoskeletal	3%	22	1.8%	1.1 – 2.6	−1.2%
Nervous system	6%	120	10.1%	8.4 – 11.7	4.1%
OB/GYN	3%	31	2.6%	1.7 – 3.5	−0.4%
Psychobehavioral	2%	64	5.4%	4.1 – 6.6	3.4%
Renal and urogenital	3%	53	4.4%	3.3 – 5.6	1.4%
Respiratory	7%	97	8.1%	6.6 – 9.6	1.1%
Toxicology	4%	131	11.0%	9.3 – 12.7	7.0%
Trauma	9%	148	12.4%	10.7 – 14.2	3.4%
Procedures	8%	467	39.1%	36.9 – 41.4	31.1%
Other	2%	864	72.4%	70.8 – 74.0	70.4%

*ABEM*, American Board of Emergency Medicine; *EM*, emergency medicine; *CI*, confidence interval; *GI*, gastrointestinal; *HEENT*, head, eyes, ears, nose, and throat; *OB/GYN*, obstetrics/gynecology.
