# Supplementary material for: An Evaluation of Emergency Medicine Core Content Covered by Podcasts
Source: West J Emerg Med. 2023 Jan 11;24(1):15–22. doi: 10.5811/westjem.2022.11.57717 (PMC9897254; doi:10.5811/westjem.2022.11.57717)
Supplement: Supplementary file 1 [file wjem-24-15-s001.docx]

**eAppendix A: List of podcasts included in our evaluation**

**From the Little paper:**

[ACEP Critical Decisions in Emergency Medicine](https://podcasts.apple.com/us/podcast/acep-critical-decisions-in-emergency-medicine/id1324257639?mt=2)

[ACEP Frontline](https://podcasts.apple.com/us/podcast/acep-frontline-emergency-medicine/id1063793120)

[AEM Early Access](http://brownemblog.com/podcasts/#aem-early-access)

[Annals of Emergency Medicine](https://www.annemergmed.com/content/podcast-archive)

[Brown EM Podcasts](http://brownemblog.com/podcasts/#aem-ET)

[CRACKCast](https://canadiem.org/category/podcast/crackcast/)

[EM Basic](http://embasic.org/)

[EM Board Bombs](https://www.emboardbombs.com/)

[EM Clerkship](http://www.emclerkship.com/)

[EM Over Easy](https://emovereasy.com/)

[EM Pulse](https://ucdavisem.com/em-pulse/)

[EM:RAP](https://www.emrap.org/)

[EMCast](https://journals.lww.com/em-news/pages/podcastepisodes.aspx?podcastid=6)

[EMCrit](https://emcrit.org/category/emcrit/)

[Emergency Medicine Cases](https://emergencymedicinecases.com/podcasts/)

[EMJ Podcast](http://feeds.bmj.com/emj/podcasts)

[EMN Live](https://journals.lww.com/em-news/pages/podcastepisodes.aspx?podcastid=7)

[EMplify](https://www.ebmedicine.net/content.php?action=showPage&pid=499&utm_source=Podcast0Postlaunch&utm_medium=Podcast&utm_campaign=Emplify)

[Everyday Medicine for Physicians](https://journals.lww.com/em-news/pages/podcastepisodes.aspx?podcastid=2)

[FOAMcast](http://foamcast.org/)

[GEMCast](https://gempodcast.com/)

[Journal of Emergency Medicine (AAEM)](https://www.aaem.org/resources/publications/podcasts/the-journal-of-emergency-medicine-audio-summary)

[Legal & Policy Issues in Emergency Medicine (AAEM)](https://www.aaem.org/resources/publications/podcasts/legal-and-policy-issues-in-emergency-medicine)

[RoshCast](https://www.roshreview.com/blog/)

[Rural EM](http://www.ruralem.org/category/podcast/)

[Talk EM](https://cme4life.libsyn.com/)

[The Skeptic’s Guide to EM](https://www.thesgem.com/category/podcast/)

**From the Social Media Index:**

[Broome docs](https://broomedocs.com/category/podcast/)

[Critical Care Practicioner](https://www.criticalcarepractitioner.co.uk/podcasts/)

[Don’t forget the bubbles](https://dontforgetthebubbles.com/podcast/)

[EMJ Club](http://emjclub.com/)

[FEMinEM](https://feminem.org/podcast/)

[Flight Bridge ED](https://www.flightbridgeed.com/index.php/podcasts)

[LITFL - Jelly Bean](https://litfl.com/podcasts/jellybean/)

[LITFL - Mastering Intensive Care](https://litfl.com/podcasts/mastering-intensive-care/)

[Paediatric emergencies](https://www.paediatricemergencies.com/podcasts/)

[PEM Blog](http://www.pemcincinnati.com/podcasts/)

[LITFL - RAGE](https://litfl.com/podcasts/rage-podcast/)

[REBEL EM](https://rebelem.com/)

[Rollcage medic](https://rollcagemedic.com/podcasts.php)

[St. Emlyn’s podcast](https://www.stemlynspodcast.org/)

[Taming the SRU](https://www.tamingthesru.com/)

[The Resus room](https://www.theresusroom.co.uk/category/podcasts/)

[Thinking critical care](https://thinkingcriticalcare.com/)

[Total EM](https://www.totalem.org/emergency-professionals)

[ToxNow](http://toxnow.org/)

**From podcast creators’ recommendations:**

[#resusTO](https://www.listennotes.com/podcasts/resusto-resusto-B8Amy4b-0UZ/)

[CORE EM](http://coreem.net/podcast/)

[Corpsey](https://tunein.com/podcasts/Science-Podcasts/Corpsey-p1271847/)

[ED ECMO](https://edecmo.org/)

[Emergency Medical Minute](https://emergencymedicalminute.com/podcasts/)

[ERCast](https://home.hippoed.com/)

[MacEmerg](https://podcasts.apple.com/ca/podcast/special-episode-dr-zain-chagla-covid-19-dept-medicine/id1450524395?i=1000469659522)

[Pediatric Emergency Playbook](https://pemplaybook.org/)
